# Effects of grinding method and particle size of wheat grain on energy and nutrient digestibility in growing and finishing pigs

**DOI:** 10.1093/tas/txaa062

**Published:** 2020-05-16

**Authors:** Jesus A Acosta, Amy L Petry, Stacie A Gould, Cassandra K Jones, Charles R Stark, Adam Fahrenholz, John F Patience

**Affiliations:** 1 Department of Animal Science, Iowa State University, Ames, IA; 2 Department of Animal Sciences and Industry, Kansas State University, Manhattan, KS; 3 Department of Poultry Science, North Carolina State University, Raleigh, NC

**Keywords:** digestibility, feed processing, grinding, particle size, swine, wheat grain

## Abstract

Feed grains are processed to improve their value in pig diets by exposing kernel contents to enzymatic and microbial action. The objective of this study was to quantify the effect of reducing mean particle size (PS) of wheat grain ground with two different grinding methods (GMs) on the apparent total tract digestibility (ATTD) of nutrients and energy in growing and finishing pigs. Forty-eight barrows were housed in individual pens for 11 d for two periods. Pigs were randomly assigned to a 3 × 2 × 2 factorial experimental design: three target mean PS of wheat grain (300, 500, and 700 µm), two GMs (roller mill and hammermill), and two body weight (BW) periods (growing period; initial BW of 54.9 ± 0.6 kg and finishing period; initial BW of 110.7 ± 1.4 kg). Diets contained one of six hard red wheat grain samples, vitamins, minerals, and titanium dioxide as an indigestible marker. Feed allowance provided 2.5 (for the two lightest pigs in each treatment) or 2.7 (for the remaining six pigs in each treatment) times the estimated daily maintenance energy requirement for each growth stage. Fecal samples were collected for the last 3 d of each period. Data were analyzed as a linear mixed model with pig as a random effect and PS, GM, and BW period and their interactions as fixed effects utilizing the MIXED procedure of SAS. Growing pigs had greater (*P* < 0.05) ATTD of dry matter (DM), gross energy (GE), N, acid hydrolyzed ether extract (AEE), and neutral detergent fiber (NDF) by lowering mean PS from 700 to 500 μm using either a roller mill or a hammermill. However, digestibility did not increase when PS was reduced from 500 to 300 μm, except for AEE (*P* < 0.05). Finishing pigs had greater ATTD of DM, GE, N, AEE, and NDF by lowering mean PS with a hammermill from 700 to 500 μm (*P* < 0.05), but it was greater for 500 μm than for 300 μm (*P* < 0.05). Using a roller mill reduced the ATTD of DM and NDF by lowering PS from 700 to 300 μm (*P* < 0.05). The ATTD of GE decreased by lowering PS from 700 to 500 μm with a roller mill (*P* < 0.05) for finishing pigs. The ATTD of N and AEE for finishing pigs were similar from 700 to 300 μm when ground by a roller mill. These data suggest that the PS that maximized digestibility for a hammermill is 500 μm for both growing and finishing pigs. However, for the roller mill, the PS resulting in the best digestibility were 500 and 700 μm for growing and finishing pigs, respectively.

## INTRODUCTION

Wheat grain is an important ingredient in swine diets in western Canada, Australia, and northern Europe, whereas its use in the United States depends on availability, location, and price. Because of its high starch content, wheat grain functions primarily as an energy source in swine diets (Zijlstra et al., 1999). The feeding value of wheat grain is estimated to be 91% to 97% of corn grain but can be enhanced with the implementation of various processing techniques (Stein et al., 2010). Reducing particle size (PS) is a common practice to improve digestibility, feed efficiency, and growth performance of swine (Hancock and Behnke, 2001). Grinding increases grain surface area facilitating contact with digestive enzymes. The resulting improvement in nutrient digestibility thus enhances the value of cereal grains in pig diets (Flis et al., 2014). The primary grinding methods (GMs) implemented in the swine industry roller milling and hammermilling. These technologies produce different particle shapes, which could have varying effects on the digestibility of wheat grain fed to pigs (Wondra et al., 1995). Whereas many experiments have investigated PS reduction of corn (Wondra et al., 1995), barley (Chu et al., 1998), and sorghum (Gieesmann et al., 1990), there are few reports on the impact of reducing the PS of wheat grain.

To maximize digestibility of wheat grain as a consequence of reducing PS, a greater understanding of the following factors are needed: 1) the effect of PS on digestibility within the range of current industry practice, 300 to 700 μm; 2) assessment of the influence of GM utilized to achieve the reduction of mean PS; and 3) the effect of the growth stage of the pig. Therefore, the experimental objective was to quantify the effect of reducing mean PS of wheat grain using two GM, either a roller mill or hammermill, on the apparent total tract digestibility (ATTD) of nutrients and energy in both growing and finishing pigs. We hypothesized that reducing mean PS would increase the ATTD of dietary components using either GM. Also, we hypothesized that the ATTD of dietary components would be greater in finishing pigs, compared with growing pigs, independent of the mean PS and the GM used.

## MATERIALS AND METHODS

Experimental procedures were approved by the Institutional Animal Care and Use Committee at Iowa State University (2-14-7731-S) and adhered to guidelines for the ethical and humane use of animals for research according to the Guide for the Care and Use of Agricultural Animals in Research and Teaching (FASS, 2010). This experiment was conducted at the Iowa State Swine Nutrition Farm (Iowa State University, Ames, IA).

### Animals Housing and Experimental Design

Forty-eight barrows (C22 or C29 dams × 337 terminal sires; PIC Inc., Hendersonville, TN) with an initial body weight (BW) of 54.9 ± 0.6 kg were randomly assigned to a 3 × 2 × 2 factorial experimental design: wheat grain ground to three target mean PS (300, 500, and 700 µm), two GMs (roller mill or hammermill) and two BW periods (growing period; initial BW of 54.9 ± 0.6 kg and finishing period; initial BW of 110.7 ± 1.4 kg), resulting in eight observations per treatment.

Pigs were individually housed in pens within a controlled environment; pens were equipped with a partially slatted concrete floor, an automatic self-feeder and a cup drinker. Pigs had ad libitum access to water. A daily feed allowance that provided 2.5 (for the two lightest pigs in each treatment) or 2.7 (for the remaining six pigs) times the estimated daily maintenance energy requirement (NRC, 2012) for each growth stage was offered in mash form, and fed twice daily at 8:00 and 16:00 h in equal sized quantities for 11 d.

The same 48 pigs were used for both BW categories. Pigs were re-randomized to treatment following the conclusion of the growing period and did not receive the same experimental treatment in both respective periods. All pigs were fed a commercial growing pig diet that was nutritionally adequate during the 45 d between the two BW periods.

### Dietary Treatments

Diets were manufactured at O.H. Kruse Feed Technology Innovation Center (Table 1; Kansas State University, Manhattan, KS) using a commercial source of hard red wheat grain that were ground according to the following specifications before mixing with other ingredients. Wheat grain was ground to 300, 500, and 700 μm, using a hammermill (model 22115, Bliss Industries, Ponca City, OK) or a roller mill (model 924, RMS Roller Grinder, Harrisburg, SD) for each PS resulting in 6 diets. The hammer mill was equipped with a 1.52-mm screen and set to a tip speed of 6,320 m/min to achieve a PS of 300 μm, a 4.06 mm screen and set to a tip speed of 6,320 m/min to achieve a PS of 500 μm, and with a 6.35 mm screen and set to a tip speed of 4,739 m/min to achieve 700 μm. The roller mill was equipped with 3 pairs of rolls: one on the top (2.36 and 2.36 corrugations/cm), one in the middle (4.72 and 5.51 corrugations/cm), and one at the bottom (6.30 and 7.09 corrugations/cm) with decreasing gaps between rolls from up to bottom to achieve a mean PS of 300, 500 and 700 μm. All diets included titanium dioxide (**TiO**_**2**_) as an indigestible marker and supplementary vitamins and minerals to meet or exceed vitamin and mineral requirements for barrows (NRC, 2012). Nevertheless, there was no inclusion of other sources of proteins, fats, or synthetic amino acids to ensure wheat grain was the only source of amino acids, carbohydrates, and energy in the diets.

**Table 1.** Ingredient composition of experimental diets^1^

Ingredient	%
Wheat grain	96.87
Monocalcium phosphate	0.48
Calcium carbonate	1.35
Salt	0.50
Vitamin premix^1^	0.20
Mineral premix^2^	0.20
Titanium dioxide	0.40

^1^There were six experimental diets in total: three particle sizes (300, 500, and 700 µm) at each of two grinding methods: hammermill or roller mill determined during the growing and finishing phases; *n* = 8 observations/treatment.

^2^Provided per kilogram of diet: 6,614 IU of vitamin A, 827 IU of vitamin D, 26 IU of vitamin E, 2.6 mg of vitamin K, 29.8 mg of niacin, 16.5mg of pantothenic acid, 5.0 mg of riboflavin, and 0.023 mg of vitamin B_12_.

^3^Provided per kilogram of diet: 165 mg Zn as ZnSO_4_, 165 mg Fe as FeSO_4_, 39 mg Mn as MnSO_4_, 17 mg Cu as CuSO_4_, 0.3 mg I as Ca(IO_3_)_2_, and 0.3 mg Se as Na_2_SeO_3_.

### Sample Collection, Physical, Chemical and Microscopy Analyses, and Calculations

Particle size distribution was measured according to the methods of Kalivoda et al. (2017) at the Kansas State University Swine Nutrition Laboratory (Manhattan, KS). Briefly, using a riffle divider and an analytical scale, an approximate 100 ± 5 g subsample of each wheat grain treatment was obtained. Subsamples were shaken with 0.5 g of dispersion agent for 15 min using a sieve shaker (model Ro-Tap RX−26, W. S. Tyler Industrial Group, Mentor, OH) furnished with 13 sieves (U.S. standard sieve Nos. 6, 8, 12, 16, 20, 30, 40, 50, 70, 100, 140, 200, and 270) and a pan equipped with sieve agitators (model SSA-58, Gilson Company Inc., Lewis Center, OH). Samples collected in the middle sieve fractions (sieves Nos. 20, 30, 40, 50, and 70) were collected for further laboratory analyses. The geometric mean diameter and geometric standard deviation were calculated according to the ANSI/ASAE S319.2 (American Society of Agricultural and Biological Engineers [ASABE], 1995) standard method.

Ten subsamples of each diet were collected at mixing, homogenized, and subsampled for analysis. Fecal samples were collected via grab sampling of fresh material from the pen floor at 0930 and 1630 h on days 9 to 11 of each test period and immediately frozen at −20 °C. Upon completion of fecal collections, fecal samples were thawed, homogenized, dried to constant weight at 65 °C (Jacobs et al., 2011) and ground in a Wiley mill through a 1-mm screen (Model ED-5, Thomas Scientific Inc., Swedesboro, NJ). Feed and sieve samples were divided into two subsamples, one ground through a 1 mm screen and the second through a 0.5-mm screen using a centrifugal mill (Model ZM1, Retsch Inc., Newton, PA). All dried samples were stored in desiccator cabinets until completion of chemical analysis.

Images depicting the topography and PS of the experimental diets were captured using field emission scanning electron microscopy (Roy J. Carter High-Resolution Microscopy Facility, Iowa State University, Ames, IA). Briefly, samples were uniformly mounted on circular aluminum stubs with double-sided carbon tape and coated with platinum to a maximum thickness of 8 nm using a high-resolution sputter coater adapted with a high-resolution thickness controller (HR 208, Cressington Scientific Instruments Ltd, Watford, UK). Samples were examined with an ultra-low voltage cold cathode field emission scanning electron microscope (Hitachi S-4800, Hitachi, Krefeld, Germany) at a voltage of 10.0 kV. Multiple representative scanning electron microscopy (SEM) pictures of each diet were captured at 50× in low magnification, and images were scaled with the associated software (4800 FE-SEM Hitachi Internal Software, Hitachi; Krefeld, Germany).

The chemical composition of sieve fractions, feed, and feces were analyzed at the Monogastric Nutrition Laboratory (Iowa State University, Ames, IA). Samples were analyzed in duplicate for dry matter (DM; method 930.15; AOAC, 2007), N, TiO_2_, and gross energy (GE) and in triplicate for acid hydrolyzed ether extract (AEE), starch, neutral detergent fiber (NDF), and acid detergent fiber (ADF). Crude protein was measured using combustion (Nitrogen Determinator; model TruMac N, Leco Corporation, St. Joseph, MI; method 990.03; AOAC, 2007) and ethylenediaminetetraacetic acid (9.57% nitrogen; Leco Corporation, St. Joseph, MI) as the standard for calibration was determined to contain 9.58 ± 0.02% N. Crude protein was calculated as N × 6.25 for feed and sieve samples. Feed and fecal samples were analyzed for TiO_2_ according to the colorimetric method of Leone (1973). Gross energy was determined using a bomb calorimeter (model 6200; Parr Instrument Co., Moline, IL). Benzoic acid (6,318 kcal/kg; Parr Instrument Co.) was used as the standard for calibration and was determined to contain 6,323 ± 2.1 kcal GE/kg.

Acid hydrolyzed ether extract was assayed using a SoxCap hydrolyzer (model SC 247) and a Soxtec fat extractor (model 255), Foss, Eden Prairie, MN (method 968; AOAC, 2007). Diet and sieve fractions were analyzed for starch using a commercially available kit (Megazyme, Wicklow, Ireland; modified method 996.11, AOAC, 1996). Feed and fecal samples were analyzed for ADF and NDF according to the modified method from Van Soest and Robertson (1980) using an Ankom automated fiber analyzer (model 2000, Macedon, NY). Hemicellulose content was calculated by subtracting ADF from NDF. Dietary analyzed nutrient composition is presented in Table 2.

**Table 2.** Analyzed chemical composition of the experimental diets (as-fed basis)^1^

	Hammermill			Roller mill		
Item	300	500	700	300	500	700
Dry matter, %	90.82	90.67	90.30	91.20	90.50	90.73
Gross energy, Mcal/kg	3.76	3.76	3.77	3.76	3.77	3.73
Crude protein, %	13.24	13.80	13.97	11.17	11.30	11.18
Acid ether extract, %	1.85	1.90	1.77	1.93	1.90	1.80
Starch, %	56.66	61.90	57.24	58.75	62.53	60.34
Acid detergent fiber, %	2.59	2.59	2.64	3.17	2.70	2.91
Neutral detergent fiber, %	9.37	9.66	9.39	10.02	9.86	9.62

^1^There were six experimental diets in total: three particle sizes (300, 500, and 700 µm) at each of two grinding methods: hammermill or roller mill determined during the growing and finishing phases; *n* = 8 observations/treatment.

The ATTD of DM, GE, N, AEE, ADF, NDF, and hemicellulose were calculated according to the equation of Oresanya et al (2008):

ATTD, % = [100 − [100 × (% TiO_2_ in feed/% TiO_2_ in feces) × (concentration of component in feces/concentration of component in feed)]].

### Statistical Analysis

Data were analyzed acoording to the mixed model:

$$\begin{array}{ll} y_{ijkl}= & \;\mu +\tau_{i}+\lambda_{j}+\theta_{k}+{\left(\tau \lambda \right)}_{ij}+{\left(\tau \theta \right)}_{ik}+{\left(\lambda \theta \right)}_{jk}\\ & +{\left(\tau \lambda \theta \right)}_{ijk}+\delta_{l}+\epsilon_{ijkl} \end{array}$$

where y_ijkl_ represents the observed value for the *l*th experimental unit within the *i*th level of PS and *j*th level of GM and *k*th level of BW category of the *l*th pig; *μ* is the overall mean; *τ* represents the fixed effect of PS (*i* = 1to 3); *λ* represents the fixed effect of GM (*j* = 1, 2); *θ* represents the fixed effect of BW category (*k* = 1, 2); *τλ* represents the interaction effect between PS and GM; *τθ* represents the interaction effect between PS and BW category; *λθ* represents the interaction between GM and BW category; *τλθ* represents the interaction among PS, GM, and BW category; *δ* represents the random effect of the *l*th pig (l =1 to 8); $\epsilon_{ijkl}$ is the associated variance as described by the model for$\;y_{ijkl}$ assuming δ ~ Ν (0, I$\sigma_{}^{2}$) and $\epsilon_{ijkl}$ ~Ν(0,$\;I\sigma_{}^{2}$), where I is the identity matrix.

The UNIVARIATE procedure of SAS version 9.3 (SAS Inst., Inc., Cary, NC) was used to verify normality and homogeneity of the residual variance from the reported model. The model was analyzed using the MIXED procedure of SAS. Differences were considered statistically significant with *P* ≤ 0.05 and trends from *P* >0.05 to *P* ≤ 0.10. Pig was the experimental unit in all instances.

## RESULTS AND DISCUSSION

**Figure 1.**
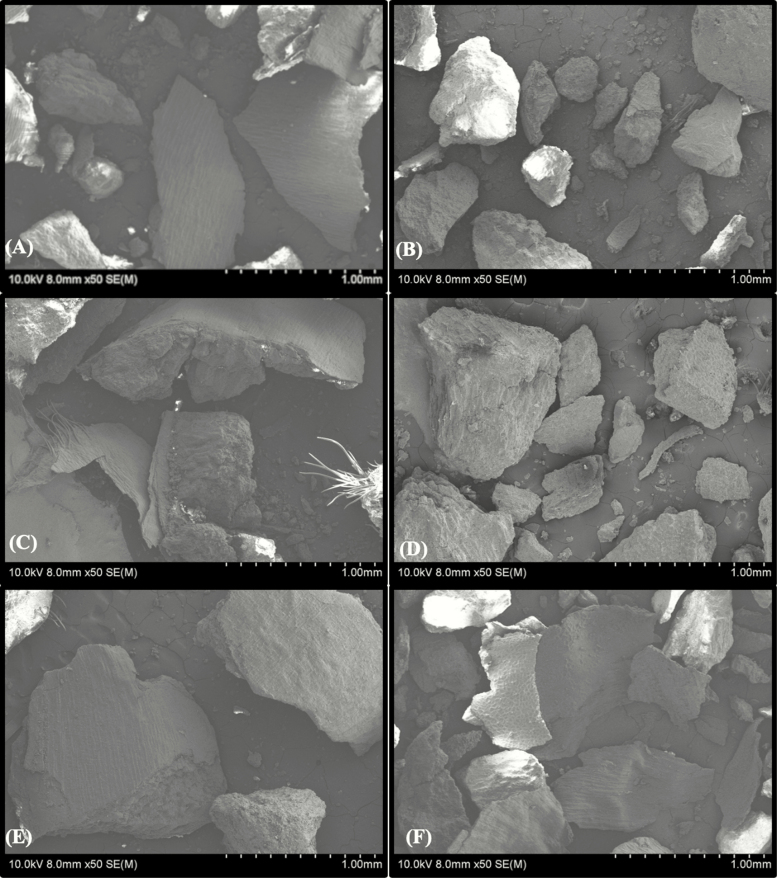
Field emission scanning electron microscopic images (×50) of wheat particles ground to a mean particle size of (A) 300 µm with a roller mill, (B) 300 µm with a hammermill, (C) 500 µm with a roller mill, (D) 500 µm with a hammermill, (E) 700 µm with a roller mill, and (F) 700 µm with a hammermill.

Grinding is a mechanical method that ruptures and reduces the wheat kernel into smaller fragments. This, in turn, results in the increased surface area through disruption of the bran and exposure of the encapsulated endosperm and germ (Rojas et al., 2016; Vukmirović et al., 2016). Disruption and alterations to the topography of the wheat kernel by grinding are distinguishable by SEM (Fig. 1). Exposing and increasing the surface area of encapsulated cellular components allows for digestive enzymes to have greater access to more valuable dietary components (i.e., starch, protein, and lipid). Fiber could potentially be fermented more easily by increasing the surface area of the bran component (Giesemann et al., 1990; Wondra et al., 1995).

### Results of PS Parameters of Wheat Grain Ground with a Hammermill or with a Roller Mill

**Figure 2.**
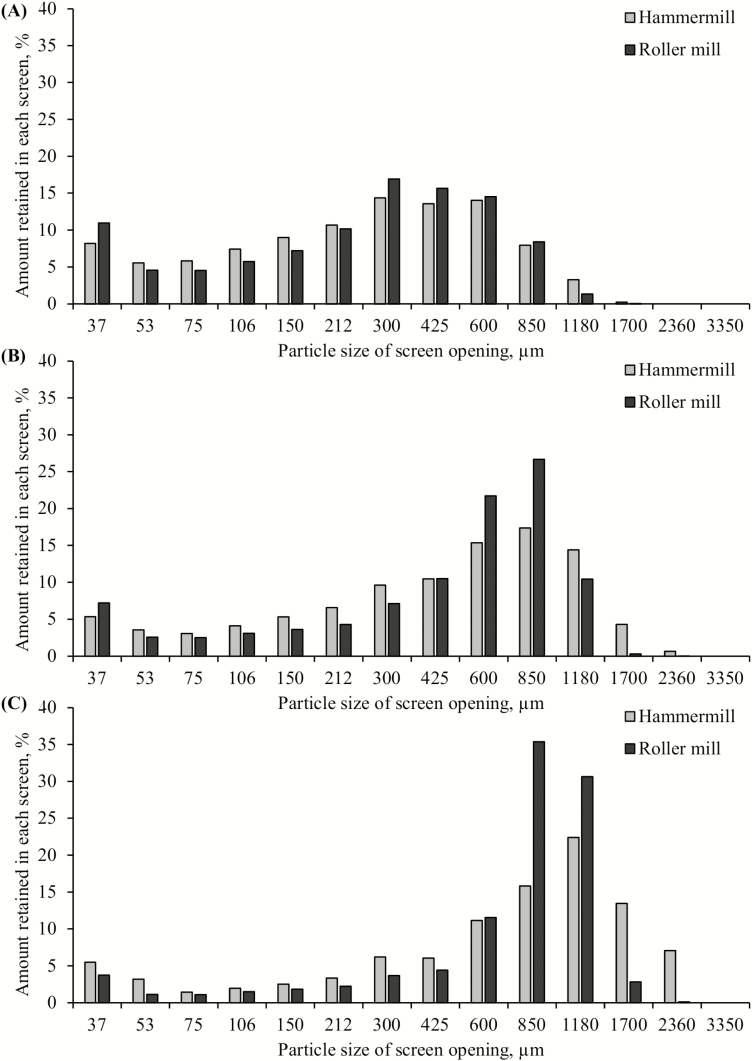
Particle size distribution of wheat, expressed as a percent of the total sample, ground using a hammermill or a roller mill to a mean particle size of (A) 300 µm, (B) 500 µm, or (C) 700 (*n* = 1 for all samples).

The determination of mean PS, as well as the sieve fraction analysis, assumes that feed particles can freely and progressively pass-through square sieve holes with a reduced sieve diameter until the width of the sieve holes is smaller than the diameter of the particle (Liu, 2009). In this study, regardless of grinding technology, mean PS were close to the targeted PSs of 300, 500, and 700 μm; mean PS for the hammermill was 278, 477, and 725 μm, and roller mill was 277, 486, and 783 μm (Table 3). Thus, the experimental model worked and provided the required samples to undertake the study. The SD of particles for the roller mill and hammermill ranged from 2.4 to 2.7 and 2.7 to 3.1, respectively. The SD of hammermilled wheat particles appears to increase with increasing PS. However, no numerical trends nor distinguishable differences were observed with those samples ground with a roller mill in PS distribution between GMs (Fig. 2). The observed PS parameters were different than those observed in a similar experiment investigating PS in corn grain (Acosta et al., 2019); in that study, the hammermill resulted in a greater SD of PS when compared with the roller mill. The absence of any difference between GM may be noteworthy since increased variation in PS can affect energy digestibility (Patience et al., 2011).

**Table 3.** Geometric mean diameter and geometric standard deviation of wheat grain ground with a hammermill or a roller mill at three different particle sizes^1,2^

	Hammermill			Roller mill		
	Targeted particle size, µm			Targeted particle size, µm		
Item	300	500	700	300	500	700
Geometric mean diameter, µm	278	477	725	277	486	783
Geometric standard deviation	2.7	2.8	3.1	2.7	2.7	2.4

^1^There were six experimental diets in total: three particle sizes (300, 500, and 700 µm) at each of two grinding methods: hammermill or roller mill determined during the growing and finishing phases; *n* = 8 observations/treatment.

^2^Variables were determined according to ANSI/ASAE S319.2 (American Society of Agricultural and Biological Engineers [ASABE], 1995) standard method for particle size analysis at the Kansas State University Swine Nutrition Laboratory.

### The Interaction Between PS, GM, and BW on Wheat Grain Nutrient Digestibility

**Figure 3.**
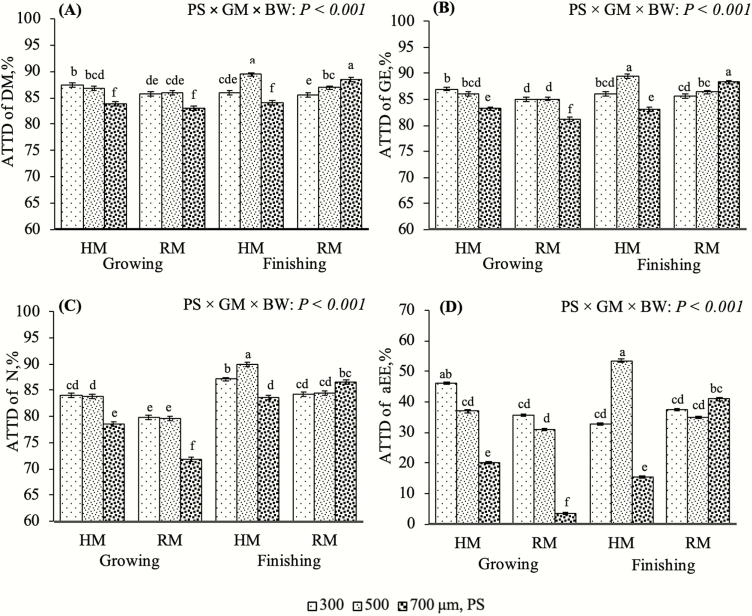
The effect of a three-way interaction among mean particle size (PS) of wheat ground with two different grinding methods [GM; hammermill (HM) or roller mill (RM)] on the apparent total tract digestibility (ATTD) of dry matter (DM; A), gross energy (GE; B), N (C), and acid ether extract (aEE; D) for two body weight periods (growing and finishing). Bars that do not share a common superscript (a–f) within a graph differ (*P* ≤ 0.05).

**Figure 4.**
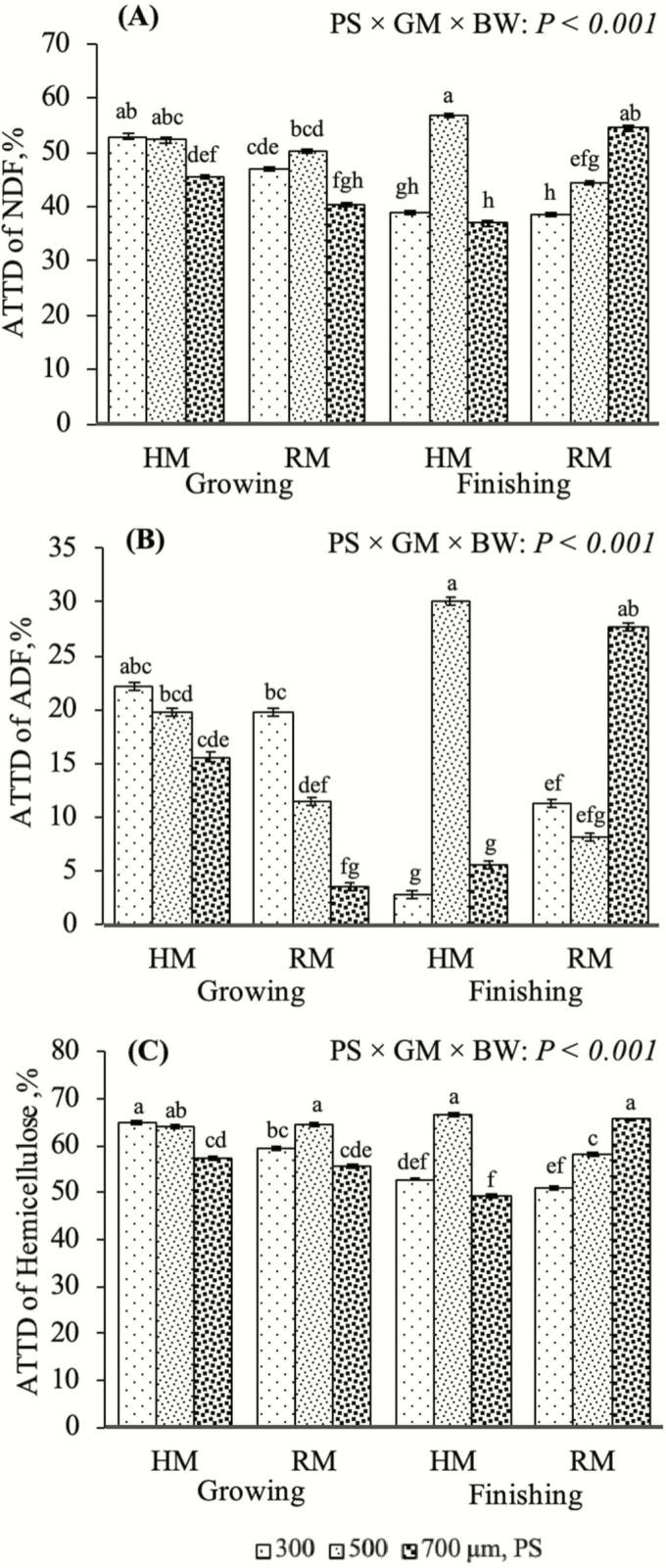
The effect of a three-way interaction among mean particle size (PS) of wheat ground with two different grinding methods (GM; hammermill (HM) or roller mill (RM)) on the apparent total tract digestibility (ATTD) of neutral detergent fiber (NDF; graph A), acid detergent fiber (ADF; graph B), and hemicellulose (graph C) for two body weight periods (growing and finishing). Bars that do not share a common superscript (a–h) within a graph differ (*P* ≤ 0.05).

Reducing mean PS of wheat grain through grinding is a common method for improving digestibility in pigs (Wu, 1985; Wondra et al., 1995; De Jong et al., 2016). In this study, it is evident that mean PS and GM can influence wheat grain digestibility differently for growing and finishing pigs. The ATTD of DM, GE, N, AEE, NDF, ADF, and hemicellulose were influenced by the interaction among PS, GM, and BW (*P* < 0.001; Figs. 3A–D and 4A–C). Reducing PS is thought to increase surface area allowing for greater interaction with digestive enzymes (Vukmirović et al., 2017). However, these data indicate decreasing PS might not increase digestibility in all circumstances. These interactions were driven by the responses observed in finishing pigs, particularly at 500 μm for the hammermill and 700 μm for the roller mill. In the finishing period, pigs had greater ATTD of all measured variables when mean PS with a hammermill decreased from 700 to 500 μm (*P* < 0.05), but it was greater for 500 than for 300 μm (*P* < 0.05). However, when using a roller mill, lowering mean PS from 700 to 300 μm decreased the ATTD of DM, NDF, and hemicellulose (*P* < 0.05). Furthermore, the ATTD of GE and ADF decreased by lowering mean PS from 700 to 500 μm (*P* < 0.05) but were similar from 500 to 300 μm (*P* > 0.05). Reducing PS with a roller mill did not affect ATTD of N or AEE for finishing pigs (*P* > 0.05).

There are limited data in the literature to explain the effects of BW and feed processing on the digestibility of wheat grain. However, it has been shown that the digestibility of nutrients is generally higher for finishing pigs than for growing pigs (Noblet and Henry, 1993). The increase in digestibility is mainly attributed to the greater development of the gastrointestinal tract, and particularly increased capability to ferment nonstarch polysaccharides (Noblet and van Milgen, 2004). Results of the current experiment suggest that, compared to growing pigs, finishing pigs digest wheat grain more effectively at a larger PS, 700 μm for the roller mill, and 500 μm for the hammermill. Therefore, finishing pigs can efficiently utilize coarser wheat grain than growing pigs. Early research suggested finishing pig growth performance was greater when fed a coarse ground wheat grain diet (1,710 μm) as compared with a finer ground diet (860 μm); these results were partially confounded as a hammermill was used for the coarse wheat grain diet, and a roller mill for the fine diet (Seerley et al., 1988). More recent experiments evaluating the PS of wheat grain in finishing pigs differ compared with those reported herein. Mavromichalis et al. (2000) reported an increase in DM and N digestibility when PS was reduced from 1,300 to 600 μm and from 600 to 400 μm in separate experiments. However, GMs could not be distinguished in their study as both technologies were used on the same sample to obtain the target final PS. De Jong et al. (2016) observed that pigs had reduced average daily feed intake and numerically lower average daily gain when fed 326 μm wheat grain from 44 to 80 kg in BW but adjusted to the finer PS from 80 to 120 kg in BW. Adaptation to PS also resulted in improved DM and GE digestibility with the 326 μm diets compared with 728 μm in finishing pigs. The work of De Jong et al. (2016) suggests a longer adaptation period could alter the results reported herein. Clearly, further research is needed to evaluate the pig’s adaptation to different PSs resulting from different grinding technologies.

These results could also be driven by the physical differences of the final ground diets. It has been reported that particles of hammermilled grains are more spherical than roller milled particles, at least for corn (Reece et al., 1985). Visual observations of the SEM images in Fig. 1 suggest that particles from hammermilled wheat grain were more spherical than the roller milled samples. Results of the current experiment suggest that the ATTD of NDF, AEE, and hemicellulose in finishing pigs is maximized at 500 μm when produced by a hammermill and 700 μm when produced by a roller mill. The shape of these particles may provide an important piece of information to help explain the differences in digestibility between GM; further research is required in this area.

Growing pigs increased the ATTD of DM, GE, N, AEE, NDF, and hemicellulose when the mean PS was reduced from 700 to 500 μm, when produced in the hammermill (*P* < 0.05; Figs. 3A–C and 4A and C). However, there was no further increase in digestibility from 500 to 300 μm (*P* > 0.05); the one exception was the ATTD of AEE, which was further increased when the PS was reduced to 300 μm (*P* < 0.05; Fig. 3D). It is also important to note that digestibility coefficients for all variables in each mean PS diameter category in the growing period were always numerically greater for wheat grain ground with a hammermill than with a roller mill. These data agree with Lahaye et al. (2008), who reported that decreasing the mean PS of wheat grain from 1,000 to 500 μm using a hammermill improved the ileal digestibility of GE and DM. However, our results were in contrast with those of Bao et al. (2016) who observed no improvements in the ATTD of DM or GE among wheat grain diets with mean PS diameters of 330, 430, 450, 470, 580, or 670 μm produced by a hammermill. These data suggest that growing pigs require a greater surface area to maximize digestibility regardless of the GM. Still, improvements may not continue past a certain threshold PS (between 500 and 300 μm). Reduced digestive efficiency of wheat grain fed at small PSs (<400 μm) could be attributed to possible alterations in gastrointestinal health and function. Finely ground wheat grain-based diets have been associated with gastroesophageal lesions, increased gastrointestinal permeability, alterations in mucosal architecture, and reduced mucin secretions (Erickson et al., 1980; Ayles et al., 1996, Brunsgaard, 1998).

### Chemical Composition of Sieve Fractions of Wheat Grain

The chemical analysis of the various sieve fractions reported herein, although novel, are simple means and should be interpreted with caution as there is no replication (Table 4). The chemical analyses of the hammermill sieve fractions suggest that particles within the median sieves, regardless of mean PS, were similar and uniform in nutrient composition and similar to the reported chemical composition for hard red wheat (NRC, 2012). It has been suggested that wheat differs from other typical energy grains (i.e., corn and sorghum) in its hammermill grinding characteristics. For example, Seerley et al. (1988), claimed the small kernel and seed coat of wheat grain permeates the blades of a hammermill more uniformly, grinding the kernel more evenly; the effect appears to increase with the use of smaller screen sizes. Because of these properties, it may be more advantageous to grind fibrous materials with a hammermill (Hancock and Behnke, 2001; Flis et al., 2014). The sieve fraction analysis and PS distribution reported herein also support the assertion of greater uniformity when using a hammermill.

**Table 4.** Analyzed chemical composition of sample fractions of ground wheat grain retained at each screen opening using either a hammermill or a roller mill, as-is basis^1^

	Wheat grain ground at 300 µm					Wheat grain ground at 500 µm					Wheat grain ground at 700 µm				
	Sieve screen opening^2^, µm					Sieve screen opening, µm					Sieve screen opening, µm				
Item^3^	212	300	425	600	850	212	300	425	600	850	212	300	425	600	850
Hammermill															
DM, %	92.4	92.2	92.1	91.7	91.9	91.6	91.6	90.8	90.6	90.0	90.5	90.7	90.9	91.2	91.2
GE, Mcal/kg	3.96	3.96	3.99	4.01	3.96	4.03	3.99	3.93	3.97	3.94	4.03	3.99	4.01	3.99	3.91
CP, %	13.2	13.7	14.4	14.9	14.3	13.6	13.9	14.6	15.2	14.7	14.2	14.2	14.8	15.9	14.5
AEE, %	1.9	2.1	2.3	2.1	1.9	2.2	2.2	2.3	2.3	1.6	2.3	2.5	2.4	2.7	1.9
NDF, %	9.5	10.4	11.1	11.9	9.5	10.1	11.0	10.8	10.3	8.8	14.1	14.1	12.5	11.1	8.1
Starch, %	58.3	56.1	57.5	56.0	57.2	56.2	55.1	53.5	52.4	57.1	54.0	53.0	49.7	50.5	55.8
Roller mill															
DM, %	91.6	91.5	91.5	91.5	91.8	92.0	91.7	91.4	90.8	90.6	93.0	93.2	93.3	91.3	91.1
GE, Mcal/kg	3.97	3.94	4.01	4.08	4.11	3.98	3.97	3.96	3.97	3.97	3.96	3.95	3.92	3.99	3.94
CP, %	11.2	11.9	13.2	13.7	13.4	10.0	10.2	10.8	12.4	12.5	10.0	10.2	10.7	12.3	11.9
AEE, %	2.1	2.5	2.9	3.0	2.8	1.9	1.9	2.1	2.7	2.4	1.8	1.9	2.1	2.7	2.3
NDF, %	5.0	7.7	13.4	20.6	26.2	7.8	8.0	8.0	10.6	13.4	10.3	11.1	9.8	10.4	10.7
Starch, %	65.9	60.5	52.2	45.5	41.2	62.6	58.4	63.2	57.5	55.4	61.4	58.3	64.0	57.7	55.4

^1^Analysis was performed in duplicate (except for NDF which was analyzed in triplicate) from the particles retained in each sieve opening.

^2^Screen sizes: 212, 300, 425, 600, and 850 µm correspond to the United States standard sieve number 70, 50, 40, 30 and 20, respectively.

^3^DM = dry matter, GE = gross energy, CP = crude protein, AEE = acid ether extract, NDF = neutral detergent fiber.

Wheat grain particles collected in the 425, 600, and 800 µm sieves ground by a roller mill to a target PS of 300 μm were relatively higher in NDF and lower in starch content compared with the 500 and 700 μm target PS, and those ground by a hammermill. Roller mills were designed to press and crush each kernel in a relatively similar manner as controlled by the space established between the rolls (Hancock and Behnke. 2001). Yet, roller mills may produce a less uniform distribution when grinding more fibrous feed ingredients, such as barley, due to the outer bran layer of the kernel (Audet, 1995; Wondra et al., 1995). The aforementioned sieve fraction chemical analysis may also indicate a similar notion for wheat; wheat has similar bran and pericarp structure to barley (Beloshapka et al., 2016).

In conclusion, these data suggest a strong interaction in the digestibility of energy and nutrients in wheat grain in pigs among mean PS, GM, and BW. Growing pigs had greater digestibilities at 500 μm compared with 700 μm, regardless of the grinding technology utilized. In finishing pigs, the greatest nutrient digestibility was at 500 μm for the hammermill and 700 μm for a roller mill. The driving factors of these interactions may be related to the geometrical composition of the particle, adaptation to the utilization of fiber with increased growth, or possible negative effects of alterations in gastrointestinal health and function when fed wheat grain at small PSs. These data suggest that it is possible to reduce the cost of electricity of grinding to finer PS. Considering nutrient and energy digestibility, mills using a hammermill can grind wheat grain to a mean PS of 500 μm to maximize nutrient digestibility throughout the grow-finish period. Feed mills using a roller mill should target 500 μm to maximize digestibility in the growing period and, if possible, increase the targeted PS to 700 μm in the finishing period.
